# Factors Associated with Engagement in University Life, and Help Seeking Attitudes and Behaviour in First Year Undergraduate Students

**DOI:** 10.3390/ijerph20010120

**Published:** 2022-12-22

**Authors:** Amelia Gulliver, Taliah Wysoke, Alison L. Calear, Louise M. Farrer

**Affiliations:** 1 Centre for Mental Health Research, National Centre for Epidemiology and Population Health, The Australian National University, Canberra, ACT 2601, Australia; 2 Medical School, ANU College of Health & Medicine, The Australian National University, Canberra, ACT 2601, Australia

**Keywords:** mental health, college students, university students, help seeking, engagement

## Abstract

Students transitioning from secondary school to university may experience unique issues that impact their mental health. There is limited research, however, on what drives first year students to seek professional help for mental health problems. There is also a current lack of knowledge about the factors that may be associated with engagement with university life in students transitioning to university, and how engagement may be related to help seeking attitudes and behaviours in a first year university population. Data (*N* = 165) were drawn from two waves (Wave 1, February 2021, and Wave 4, June 2021) of a longitudinal study of Australian university students commencing study for the first time, which included measures of engagement, belonging, stigma and help seeking intentions and behaviours. The results showed that students with higher levels of depression stigma prior to commencing university at Wave 1 had less positive attitudes towards help seeking at Wave 4. Students had increased odds of seeking help for a mental health problem in Wave 4 if they had moved away for university, reported higher levels of mental health literacy or willingness to disclose, had lower levels of engagement with university life and were experiencing higher levels of general psychological distress. Students experiencing higher thwarted belongingness were also found to have lower levels of engagement with university at Wave 4. Both thwarted belongingness and stigma were found to be associated with engagement with university and help seeking behaviours and should be examined further.

## 1. Introduction

### 1.1. Mental Health Problems in University Students

Starting university symbolises an important transition in many young people’s lives, marking the end of adolescence and the beginning of young adulthood [1]. This period is frequently accompanied by other significant changes in students’ lives, including an alteration in living circumstances, and the establishment of new friendships and support networks [1,2,3]. Many students have difficulty with this transition, with up to 20% of Australian first year students not completing their first year of university [4]. The age at which most students begin their first year of university also makes them vulnerable to poorer mental health, as the onset of most mental disorders is typically before 24 years of age [1]. International research suggests that common mental illnesses, including anxiety and depression, are higher in university cohorts when compared to same-aged peers in the general community, suggesting there are other additional factors that may put students at risk [5]. These may include academic pressures and greater external expectations on performance, financial pressures of living, being from a rural or regional location, being part of a racial minority, and being gender diverse or a member of the LGBTQIA+ community [1,5]. Based on these factors, first year university students are at a significant risk of mental ill health.

The prevalence of mental disorders and generalised psychological distress in university students is high globally. For example, an international study of 14,000 students spanning 19 universities and eight countries found that 35% of students met the diagnostic criteria for one or more mental disorders [6]. A 2016 Australian study by Farrer et al. [7] found that the prevalence of major depressive disorder and generalised anxiety disorder in university students was 7.9% and 17.5%, respectively. Further, a systematic review by Sheldon et al. found that the pooled prevalence of depression in university students was 25% (95% CI 17%, 35%) and suicidal ideation was 7.6% [8]. In a UK study by Russell and Shaw [9], 6.5% of university students were found to have mild social anxiety, 6.7% had marked anxiety and 2.5% had severe social anxiety. Generalised psychological distress is also common. Australian studies have reported that 65% of university students experienced high to very high levels of emotional distress [10].

### 1.2. Help Seeking

Despite the high rates of mental disorders in this group, students frequently do not seek help [11]. The literature surrounding help seeking behaviour suggests that increased mental health literacy (an individual’s ‘knowledge’ about mental disorders that aids in their recognition, management and prevention) may lead to increased help seeking behaviour in university students [12]. However, studies have shown that university students may have limited mental health literacy skills, therefore leading to poor help seeking attitudes, and a lack of both the intention to seek help and actual help seeking behaviour [13]. Mental health literacy also encompasses help seeking knowledge, or an understanding of what formal sources (psychologists, psychiatrists, counsellors, general practitioners, etc.) are available, the processes surrounding help seeking and knowing when to seek help [11]. Poor help seeking knowledge reduces help seeking behaviour, presumably because individuals are unable to recognise symptoms of mental illness and are unaware of available treatments [14]. Therefore, for help seeking behaviour to be actioned, a person must have both high help seeking knowledge and intentions to disclose. Intentions to disclose by university students are strongly influenced by internal beliefs about stigma, fear of potential repercussions socially or academically and feelings of social isolation and lack of belonging [15,16]. Stigma has been cited as the single most common barrier to help seeking behaviour and disclosure amongst university students [17]. Stigma towards mental illness can be both internal, towards oneself or external towards others, and has been associated with negative help seeking attitudes and reduced help seeking behaviour [18]. Studies have shown that lack of disclosure and avoidance of help seeking among students is associated with poorer long-term outcomes and increased social isolation [19]. Thus, it is critical to determine what factors influence students help seeking attitudes and to consequently perform help seeking behaviours [16].

### 1.3. Engagement with University and Belongingness

Feelings of social isolation and lack of belonging are negatively associated with help seeking attitudes [15,16]. Previous international studies have shown that connectedness with university, belonging and group identity have a significant impact on attitudes towards help seeking and subsequent help seeking behaviours [16]. Social Identity Theory (SIT) proposes that identity is a social construct, wherein we define ourselves by our membership to specific groups [20]. Having a strongly defined sense of group identity plays a major role in an individual’s sense of belongingness [20]. These shared identities and feelings of belonging have been shown to improve a range of psychological and behavioural outcomes, such as elevated self-esteem, social competence, resilience and reduced loneliness [21,22]. Cameron et al. [23] explored the relationship between social identity and psychological wellbeing and found that group efficacy (a group’s perceived capability to perform) positively predicted psychological wellbeing. It was therefore suggested that group efficacy based on strong feelings of group identity motivated individuals to perform behaviours that improved their ability to contribute and perform within the group [22]. These behaviours included actions that improved their own mental wellbeing, such as disclosing of mental ill health and accessing psychological services [22]. A key influencing factor on group identity is the time spent amongst those who also identify with that shared group [24]. When looking at university as a shared group, a stronger level of group identity is arguably associated with stronger associations with campus life and higher engagement with academic, recreational and social activities at university [24]. Aspects that influence these include living on campus or level of engagement with university committees, academic clubs and student organisations [24]. When considering the role of group identity and help seeking intentions and behaviour in university students, A study in Ireland by Kearns et al. [16] found that high levels of group identification were significant in predicting students stigma towards help seeking at the university and actual help seeking behaviour, with every additional unit on the group identification scale there was a 0.10 increase on the stigma help seeking scale. Walton and Cohen [25] similarly found that their social-belonging intervention reduced feelings of adversity, improved feelings of subjective happiness and reduced mental and physical ill-health presentations to doctors among US college students. However, there have been few studies in Australia that have examined the relationships between engagement with university life, sociodemographic factors, and help seeking for mental health problems.

### 1.4. Impacts of COVID

The COVID-19 pandemic has had a significant impact on tertiary education, particularly in relation to face-to-face teaching, in that it required almost all Australian campuses to pivot to online delivery for all lecture, tutorial and seminar teaching [26]. The closure of campuses during lockdowns also significantly impacted extracurricular activities such as clubs, student organisations, and sporting teams which were no longer sanctioned to run in person [26]. On-campus student housing in Australia was also significantly affected. During the peak of COVID-19 isolation in 2020, students were either forced to single room isolation or asked to find alternate accommodation due to risk of COVID-19 exposure and transmission [26]. Studies conducted both within Australia and globally have demonstrated that these factors contributed greatly to social isolation, increased anxiety, stress and loneliness among students [27]. To our knowledge, there are no studies that have examined the impacts of online learning as was implemented during the COVID-19 pandemic on the mental health and wellbeing of Australian university students and their help seeking attitudes and behaviours.

### 1.5. Aims

The primary aims of the current study were to explore and identify factors predicting and associated with (1) engagement in university life, (2) help seeking attitudes and (3) help seeking behaviour in a longitudinal survey of Australian undergraduate university students commencing university for the first time. The study was partly exploratory, particularly for factors predicting and associated with engagement in university life; however, our hypotheses based on the literature were that we expected (1) higher levels of stigma prior to commencing university at Wave 1 to predict lower (less positive) help seeking attitudes and fewer help seeking behaviours at Wave 4, and (2) lower levels of thwarted belongingness and higher engagement in university life at Wave 4 to be associated with higher (more positive) help seeking attitudes and greater help seeking behaviours at Wave 4.

## 2. Materials and Methods

### 2.1. Participants

The projected sample size for the Wave 1 survey was 1100 undergraduate students, which accounted for an attrition rate of 40% by Wave 4, and was based on an approximate population size of 400,000 commencing first year students in 2021 (margin of error of 5% and 99% CI). However, the final full sample comprised 340 students who provided data for at least one wave. The current study reports data only from students (*N* = 165) who completed the baseline demographic data, and the outcome variables of interest, which were help seeking attitudes, behaviour, and engagement in university life at Wave 4.

Participants were 165 undergraduate students attending university for the first time during Semester 1 2021 in Australia. Participants were invited to complete an online survey via paid social media (Facebook) advertising in February 2021. Facebook advertisements targeted 18 to 25 year olds and included the following wording paired with a range of images of students, and a link to the study webpage: “Are you starting university in Semester 1? ANU researchers are looking for first year students for a survey study”. Facebook advertisements ran continuously between 4 February and 3 March 2021. Recruitment emails were also sent to university administrative staff directly to distribute to students, and Facebook messenger posts with links to the advertisement to distribute on their Facebook pages were sent to the administrators of university-affiliated student Facebook groups, such as student associations.

### 2.2. Procedure

Students completed four voluntary online surveys (10–20 min each) delivered at 6-week intervals during the first 6 months of their first year of university study. The baseline survey was completed prior to the start of the semester. Students who completed the baseline survey were emailed reminders to complete the survey at each subsequent wave. They received an AUD 15 e-gift card for completing all survey waves. Wave launch dates were as follows: Wave 1 (baseline, February 2021), Wave 2 (March 2021), Wave 3 (April/May 2021), and Wave 4 (June 2021).

### 2.3. Measures

#### 2.3.1. Demographic Characteristics

We collected the following demographic characteristics at Wave 1: age (in years); gender (male/female/other); student status (international/domestic); intentions to live in university residential housing (yes/no); relocation from home to attend university (yes/no); state or territory location of university campus (NSW/QLD/ACT/VIC/ SA/WA/TAS/NT); and approximate proportion of online vs. in-person classes (1 = 0%, 2 = 25%, 3 = 50%, 4 = 75%, 5 = 100%). Further descriptive characteristics collected were ethnicity, and whether respondents self-described as being from a low socio-economic background. We collected a range of variables at each wave of data collection (see Table S1, Supplementary File S1), which were not included in the current analysis as they were not critical to the study’s primary aims. For the full survey we selected measures primarily based on previous literature where established relationships had been found, and critical variables of interest such as mental health symptoms or engagement in university life that may be expected to vary over time given the longitudinal design. In addition, because of the timing of the survey, some measures were also selected specifically based on the context (e.g., the measurement of online learning during the COVID-19 pandemic). Measurement selection for each wave was primarily pragmatic, with variables employed when it made most conceptual sense to measure them, whilst also balancing participant burden at each wave.

#### 2.3.2. General Psychological Distress

We used the Distress Questionnaire-5 (DQ5, [28]) to assess general psychological distress. The measure comprises five items assessing the frequency of a range of distressing situations, thoughts, and feelings over the past 30 days on a 5-point scale (1 = Never, 2 = Rarely, 3 = Sometimes, 4 = Often, 5 = Always). Item scores are summed and total scores range from 5–25. Higher scores indicate higher severity of general psychological distress. A cut-point of ≥14 indicates a high level of distress and a greater likelihood of experiencing a mental disorder [28]. The DQ5 has shown high internal consistency (α = 0.86–0.91) and external validity in previous studies of adults [28,29]. The internal consistency was very good in the current sample at baseline (α = 0.87).

#### 2.3.3. Personal Stigma

The personal stigma sub-scale of the Depression Stigma Scale (DSS [30]) was used to assess stigmatising attitudes towards depression. Respondents are asked to indicate their level of agreement with each of nine statements about depression using a 5-point Likert scale (strongly disagree = 0, strongly agree = 4). Example items include “People with depression could snap out of it if they wanted” and “Depression is not a real medical illness”. Scores are summed, with total scale scores ranging from 0–36, and higher scores indicate higher levels of personal stigma about depression. The DSS has demonstrated validity and reliability in adults [30,31], with previous study internal consistency ranging from α = 0.76–0.82 [31,32,33]. The internal consistency was very good in the current sample at baseline (α = 0.85). We used DSS scores from Wave 1 to examine the effect of pre-university levels of stigma on help seeking attitudes and behaviour at Wave 4.

#### 2.3.4. Thwarted Belongingness and Engagement with University Life

We used the thwarted belongingness subscale of the Interpersonal Needs Questionnaire to assess feelings of belonging over the past 2 weeks (INQ-15) [34]. The INQ thwarted belongingness was originally developed to assess suicide risk, based on Joiner’s interpersonal-psychological theory of suicidal behavior [35]. The scale comprises 9 items such as “I rarely interact with people who care about me” and ”I am fortunate to make many caring and supportive friends”, which are rated on a 7-point scale, ranging from 1 ‘Not at all true for me’ to 7 ‘Very true for me’. After reverse scoring positively worded items, a total score is determined by calculating the mean of the scores, with higher scores indicative of greater levels of thwarted belonging (i.e., lower perceived belongingness). Previous studies have demonstrated the scale’s reliability and validity in adults [34], with internal consistency ranging from α = 0.85–0.89 [36,37]. The scale had very good internal consistency at baseline in the current sample (α = 0.90).

To capture respondents’ engagement with university life, we purpose-designed an item for the current study. Engagement was measured using a single item: “How engaged are you with university life outside of your studies?” A five-point response scale was used with the following options: 1 = Not at all (only attend classes), 2 = Somewhat, 3 = Moderately, 4 = Highly, 5= Extremely highly (involved in leadership activities within student associations, clubs, societies, halls, sports, etc.). Scores ranged from 1 to 5, with higher scores indicating higher engagement with university life.

#### 2.3.5. Help Seeking

Attitudes to seeking professional psychological treatment were assessed using five items with updated language and wording [38] from the short form of the Attitudes Towards Seeking Professional Psychological Help scale (ATSPPH-SF [39]). We have found reverse-scored items on the 10-item scale did not load well on a single factor [32]; thus, these items from the original scale were removed, with the remaining five items retained (items 1, 3, 5, 6, 7). Each of the five items asked respondents to rate their view on statements about psychological treatment using a 4-point Likert scale (Disagree = 0, Agree = 3). Scores on the 5-item abbreviated scale range from 0–15, with higher scores indicating more positive attitudes towards seeking professional help. An example item included “If I was having personal or emotional problems, the first thing I would do is seek professional help”. The original 10-item scale has previously shown sound psychometric properties including validity and reliability [38,39,40], and internal consistency for the modified 5-item scale has been noted as α = 0.77 in previous research involving adults [32]. The internal consistency was good in the current sample at Wave 1 (α = 0.71).

We also used two items related to help seeking that were purpose-designed for the current study. One assessed perceived help seeking knowledge: “Would you/do you know how and where to seek help if you were experiencing a mental health problem at university?” (yes/no) and the other assessed disclosure willingness: “Hypothetically speaking, if you were having any problems with your mental health at university, would you disclose this to anyone?” (yes/no).

Help seeking behaviour was measured by the proportion of participants who reported seeking help for a personal or emotional problem from any formal source in the last month, using the Actual Help Seeking Questionnaire (AHSQ [41,42]). The formal sources of help were (1) private or public psychologist, psychiatrist, or counsellor, (2) on-campus psychologist, psychiatrist, or counsellor, or (3) Doctor/GP (general practitioner).

### 2.4. Data Analysis

We used two waves of data from the original survey to assess if demographic characteristics and attitudes at Wave 1 (prior to commencing study, February 2021) predicted, and student mental health and attitudes at Wave 4 (mid-first year, June 2021) were associated with engagement with university, help seeking attitudes, and help seeking behaviour at Wave 4. In general, the analyses used cross-sectional data from Wave 4 (except demographic data collected at Wave 1) to examine associations between predictor and outcome variables. We carefully considered which measures to use, and from which wave in each analysis according to what made logical and temporal sense. For example, because the help seeking behaviour measure only captured help seeking during the past month, it made sense to include predictors (e.g., distress, belongingness) from the same Wave given Waves 1 and 4 were collected 4 months apart. We used a measure of willingness to disclose a mental health problem at university at Wave 1, to examine the effect of these attitudes prior to commencing study and being exposed to the university context, whereas we used a measure of self-perceived knowledge of how and where to seek help at Wave 4, with the expectation that students would likely have greater knowledge of health care options after commencing university, which may have impacted their help seeking behaviour at that time (Wave 4). Both these factors could also be important modifiable targets if they were found to significantly impact on help seeking. To identify significant predictors and associated factors for our dependent variables at Wave 4, we conducted two multiple linear regressions for engagement with university life and help seeking attitudes, and a logistic regression for help seeking behaviour. All variables were entered into the regression models simultaneously.

### 2.5. Variables

Variables included for Analysis 1 on engagement with university life were: age, gender, student status (international), living situation (on campus), moved for university (yes), proportion of online classes, INQ-Belongingness and DQ5-General Psychological distress.

Variables included for Analysis 2 on help seeking attitudes were; age, gender, student status (international), living situation (on campus), moved for university (yes), proportion of online classes, engagement with university life, INQ-Belongingness and depression stigma (DSS).

Variables included for Analysis 3 on formal help seeking behaviour were: age, gender, student status (international), living situation (on campus), moved for university (yes), proportion of online classes, engagement in university life, INQ-Belongingness, depression stigma (DSS), perceived knowledge of how to seek help, willing to disclose at university and DQ5-General Psychological Distress. Data analyses were conducted with SPSS v27 (IBM Corp, Chicago, IL, USA).

## 3. Results

Table 1 presents the sample demographic information and the key variables used in the analysis. The sample comprised a majority of female university students; and Victoria (VIC), New South Wales (NSW), and the Australian Capital Territory (ACT) were the most represented states/territories. Around half of students identified as having a European/Caucasian ethnicity (*n* = 93; 56.4%), with the remainder being from South Asian (*n* = 12; 7.3%), South East Asian (*n* = 24; 14.5%), East Asian (*n* = 26; 15.8%), Aboriginal and/or Torres Strait Islander (*n* = 3; 1.8%), or other (*n* = 7; 4.2%) backgrounds. A total of 41 students (24.8%) self-described as being from a low socio-economic background.

### 3.1. Analysis 1: Engagement with University Life

Table 2 presents the linear regression analysis assessing the exploratory analysis investigating predictors and factors associated with engagement with university life. In this model overall, the factors accounted for 11% of the variance for engagement, *R*^2^ = 0.11, adjusted *R*^2^ = 0.59, *F*(9, 155) = 2.15, *p* = 0.029. Higher levels of thwarted belongingness (i.e., lower perceived belongingness) at Wave 4 was associated with lower levels of engagement with university life at Wave 4, while those who lived on campus had higher engagement with university life at Wave 4.

### 3.2. Analysis 2: Help Seeking Attitudes

Table 3 presents the linear regression analysis assessing predictors and factors associated with help seeking attitudes. For this model overall, the factors accounted for 13% of the variance for engagement, *R*^2^ = 0.13, adjusted *R*^2^ = 0.76, *F*(10, 154) = 2.35, *p* = 0.013. Hypothesis 1 was partly supported, with higher levels of depression stigma at Wave 1 predicting lower help seeking attitudes at Wave 4 (*p* < 0.001*); however, Hypothesis 2 was not supported as engagement in university life and thwarted belongingness at Wave 4 were not associated with help seeking attitudes at Wave 4.

### 3.3. Analysis 3: Help Seeking Behaviour

Table 4 presents the logistic regression analysis assessing predictors and factors associated with help seeking behaviour. The logistic regression model was significant, *χ*^2^(13) = 48.63, *p* < 0.001. Overall, the model explained 41% (*R*^2^) of the variance in help seeking behaviour. Relocation for university, perceived help seeking knowledge, willingness to disclose mental health problems at university, lower levels of engagement in university life, and higher general psychological distress were associated with increased odds of seeking help from at least one formal source at Wave 4. Hypothesis 1 was not supported as stigma at Wave 1 did not significantly predict help seeking behaviour at Wave 4. Hypothesis 2 was partly supported with only higher engagement in university life but not lower thwarted belongingness significantly associated with greater help seeking behaviour.

## 4. Discussion

The current study sought to investigate factors predicting and associated with engagement in university life, and help seeking attitudes and behaviour in undergraduate students commencing university for the first time. The findings are discussed below and contextualised within the context of previous international research.

We found that those with higher current levels of thwarted belongingness (i.e., lower perceived belonging) had lower concurrent levels of engagement with university life. This finding supports previous research conducted in the US by Knifsend [43] who found that levels of engagement based on hours spent on specific activities (e.g., sports, social organisations, cultural groups, etc.) were associated with higher levels of belonging and lower levels of loneliness and social anxiety in university students. Another study conducted in the Netherlands during the COVID-19 pandemic by Versteeg et al. [44] found that students’ sense of academic belonging and perceived social integration were both significantly associated with engagement in university. Our finding adds to this research, suggesting that there may be protective effects on social wellbeing and mental health for students who participate in university groups or activities. Though, it could also be that those who perceive a sense of belonging are more likely to engage. The only demographic factor associated with engagement in our study was living on campus. This is consistent with research by Graham et al. [45], which showed in their survey of first year US college students that, living on campus had a small positive effect on engagement with university and extracurricular activities.

A key aim of this research was to examine factors associated with help seeking in first year university students. We found that higher levels of stigma when commencing university (Wave 1) significantly predicted less positive help seeking attitudes at the end of semester 1 (Wave 4). This supports recent research from a study of university students in Portugal [46], which found, using the longer versions of the same measures as the current study, that depression stigma was significantly and negatively associated with help seeking attitudes in this group. Stigma is clearly a pivotal factor in influencing help seeking attitudes, but there is still debate about the role of internal stigma versus perceived public stigma [47]. The measure of stigma we used examined internalised personal stigma, specifically towards depression [30]. Lally et al. [47], examined both forms of stigma within a university student population in Ireland and found that while there was no relationship between perceived public stigma and help seeking intentions, personal stigma was significantly associated with a decreased likelihood of future help seeking intentions. Moreover, Golberstein et al.’s [48] study of US university students also found that whilst perceived public stigma appeared to influence younger university students’ perceived need for seeking help (in those with probable depression or anxiety), it did not impact or predict actual use of mental health services (help seeking behaviour) [48]. Overall, this suggests the possibility that personally stigmatising views, such as thinking that depression is a sign of personal weakness or not a real medical illness, may have more impact on ones’ own help seeking, than a more global perceived assessment of what others believe.

We also found that formal help seeking behaviour at Wave 4 was higher among those who simultaneously perceived they had sufficient knowledge of how to seek help, those with higher willingness to disclose a mental health problem at university, and those with higher general psychological distress. Help seeking attitudes and behaviour have been found to be significantly related [49]; but this relationship remains complex. Specific factors such as an individual’s favourable attitudes towards mental health services, their perceived resources, mental health literacy, and those with a perceived need to access services are more likely to turn help seeking intentions into help seeking behaviours [49]. Our study supports this, as we found that those with greater knowledge about where to seek help, those with a greater willingness to disclose, and those with a greater perceived need (i.e., higher distress), actually sought professional help. Personal stigma was not significant in the final model; given that stigma was the only predictive factor on help seeking attitudes in the current study, this finding is unexpected. However, prior research has also found mixed results. For example, stigma was found not to be a significant factor in predicting delays in help seeking behaviour in a study of university students in Japan [50]. Perhaps, more direct factors such as willingness to disclose, and perceived knowledge of how and where to seek help may have been more influential in accounting for help seeking behaviour in our study. Golberstein et al. asserted [48], that levels of mental health literacy may be more important than stigma in the student population. Although we did not explicitly measure it, willingness to disclose may also be acting as a proxy for lower levels of self-stigma; thus, those not embarrassed or ashamed about their own experience of mental health problems may be more willing to seek help, and in turn more likely to enact this behaviour.

Those with lower levels of engagement with university life, and those who had moved away from home to attend university were also more likely to seek help at Wave 4. This, and our finding that thwarted belongingness was not significantly related to seeking help, was not supportive of Hypothesis 2, which was based on previous work by Cameron et al. [23] using social identity theory. However, this may reflect that being more socially engaged in university life is protective, or it may indicate that reduced informal social networks and connections mean that students may go to formal sources first, as they may not have accessible informal sources or sufficient peer support. Alternatively, greater engagement with university life might be associated with lower levels of help seeking, due to stigma [17], because students may not want others to know they are seeking help. For example, if students are well connected at university and in many social or student groups, help seeking might be harder to conceal, or may be perceived by the individual to have potentially negative impacts on their social position and potential suitability for high profile or leadership roles. In support of this, Kearns [16] found that in Ireland, students who most identified with their university had the most difficulty in performing help seeking behaviours from those affiliated with the institution. In addition, relocating for university was another significant predictor of help seeking behaviour in students in the current study. This may indicate that students who have left their home to attend university may experience poorer mental health, supporting previous research [2], thus, experiencing a greater need for help seeking. Alternatively, perhaps students living away from home have a greater willingness to seek help due to reduced social support networks. Correlations between these factors were briefly investigated in the current data set, but were not found to be significant; however, it is likely that these relationships are interrelated and highly complex, requiring further research to explore the association between lower engagement in university life, moving away from home, and greater help seeking behaviour.

### 4.1. Implications

This study highlights the complex connections that exist, particularly within the space of help seeking attitudes and behaviours. However, there are some potential ways forward. Given that engagement with university life may be impacting on student perceptions of belongingness, it might be of use to target the encouragement of engagement in student groups and activities specifically to those who do not live on campus. Further, whilst personal stigma appears to be a strong factor in influencing students’ help seeking attitudes, it is possible that other more direct factors such as knowledge about where and how to seek help may be more important in influencing actual help seeking behaviour. Mental illness is known to reduce academic performance in first-year university students [51]. Noting that only 60% of students at Wave 4 had self-perceived knowledge of how and where to seek help, improving mental health literacy in this group is a critical but potentially simple target for improving help seeking rates in university students, which may have flow on effects for improving educational outcomes in higher education. Finally, whilst the relationship between help seeking behaviours and student engagement in university and demographic factors is likely complex, there may be value in increasing access to social support for students who may have limited social support networks in the first year of university, such as those who have moved away from home.

### 4.2. Limitations

The current study has several limitations. Firstly, the study design was only partly longitudinal, meaning that some of the findings between factors and outcomes are primarily associations in the cross-sectional data. In addition, the overall survey measured a large range of variables; however, due to the scope of the current paper we only included certain measures; thus, there may be other important predictors of engagement, and help seeking attitudes and behaviours in this group that were not captured. Another limitation is that the study fell short of the expected sample size, and had a relatively poor retention rate, with only 165 participants from the full baseline sample of 340 with data available at both waves. We note this did not impact on statistical power for the current analysis (all analyses achieved power > 0.90); however, it may have resulted in a less representative, and potentially biased sample. The participants who were retained were predominantly female (72.1%), which is typical of self-selected online mental health surveys [28]. However, this means that differences in social structure and interpersonal relationships between genders could have accounted for novel findings within the research. Another limitation of the study was that the survey was performed during the height of the COVID-19 pandemic (Wave 1: February 2021, Wave 4: June 2021), which may have impacted the findings. It is unknown the extent of impact that COVID-19 had on the survey, but it should be noted that findings potentially relevant to the COVID-19 context, that were not found in the current study to be significant predictors of engagement in university life (such as relocation for university, international student status and proportion of online classes), were found to be significant in studies conducted prior to the pandemic [45].

## 5. Conclusions

The results of this study indicate that first year undergraduate students with higher levels of stigma prior to commencing university (Wave 1) had less positive attitudes towards help seeking at Wave 4 (mid-year). Wave 1 factors that were found to be significant in predicting formal help seeking behaviours at Wave 4 were relocating for university, and willingness to disclose a mental health problem at university, whilst factors associated with help seeking mid-year were perceived knowledge of how and where to seek help (mental health literacy), lower levels of engagement with university life, and high levels of general psychological distress. Thwarted belongingness was found to be associated with lower levels of engagement with university life, whilst living on campus was associated with greater levels of engagement with university life. The current study indicates the need to further evaluate university mechanisms to increase engagement and reduce mental health stigma, ultimately both with the goal to increase help seeking behaviours amongst students.

## Figures and Tables

**Table 1:** Sample demographic information (*N* = 165).

	Total
Independent variables	
Age (years), *M* (*SD*)	18.68 (1.60)
Gender, *n* (%)	
Male	36 (21.8)
Female	119 (72.1)
Prefer to describe as: Non-binary (*n* = 6) genderfluid (*n* = 2) genderqueer (*n* = 1)	9 (5.5)
Prefer not to say	1 (0.6)
State, *n* (%)	
NSW	41 (24.8)
QLD	15 (9.1)
ACT	26 (15.8)
VIC	53 (32.1)
SA	12 (7.3)
WA	16 (9.7)
TAS	2 (1.2)
NT	0 (0)
Other demographics, *n* (%)	
International student (W1)	7 (4.2)
Plan to live in university residential housing (W1)	27 (16.4)
Moved away from home to attend university (W1)	52 (31.5)
Willingness to disclose a mental health problem at university (W1)	92 (55.8)
Self-perceived knowledge of how and where to seek help (W4)	100 (60.6)
Proportion of classes online (W4)	
1 = 0%	15 (9.1)
2 = 25%	29 (17.6)
3 = 50%	48 (29.1
4 = 75%	49 (29.7)
5 = 100%	24 (14.5)
Overall score % classes online	3.23 (1.17)
Mental health related measures	
Distress (DQ5) (*M, SD*) (W4)	12.7(4.89)
Distress (DQ5) (≥14) *n* (%) (W4)	76 (46.1)
Depression stigma (DSS) (*M, SD*) (W1)	8.07 (6.01)
Thwarted belongingness (INQ) (*M*, *SD*) (W4)	3.40 (1.27)
Dependent variables	
Engagement with university life outside of studies (W4) (*M, SD*)	2.12 (1.02)
Help seeking attitudes (ATSPPH-SF) (W4) (*M, SD*)	8.96 (3.12)
Help seeking behaviour-sought help from formal sources (W4) *n* (%)	31 (18.8)

Note: W1 = Wave 1, W4 = Wave 4; NSW = New South Wales, QLD = Queensland, ACT = Australian Capital Territory, VIC = Victoria, SA = South Australia, WA = Western Australia, TAS = Tasmania, NT = Northern Territory.

**Table 2:** Linear regression model for engagement in university life at Wave 4 (*N* = 165).

	Estimate	S.E.	*t*	*p*
Intercept	2.892	1.187	2.437	0.016
Age (in years) (W1)	−0.016	0.062	−0.256	0.798
Gender (female) (W1)	0.207	0.194	1.067	0.288
Gender (other/prefer not to say) (W1)	−0.123	0.377	−0.327	0.744
Gender (male) (W1)	(Ref)			
Student status (international) (W1)	0.700	0.478	1.000	0.145
Living situation (on-campus) (W1)	0.584	0.268	2.179	0.031 *
Relocated for university (yes) (W1)	−0.055	0.215	−0.253	0.800
Proportion of online classes (W4)	−0.076	0.067	−1.126	0.262
INQ-Belongingness (W4)	−0.150	0.075	−2.008	0.046 *
DQ5-General Psychological Distress (W4)	0.002	0.020	0.105	0.916

Note: * *p* > 0.05; Ref = Reference category; W1 = Wave 1; W4 = Wave 4.

**Table 3:** Linear regression model for help seeking attitudes at Wave 4 (*N* = 165).

	Estimate	S.E.	*t*	*p*
Intercept	5.118	3.680	1.391	0.166
Age (in years) (W1)	0.268	0.187	1.433	0.154
Gender (female) (W1)	0.552	0.606	0.912	0.363
Gender (other/prefer not to say) (W1)	0.113	1.165	0.097	0.923
Gender (male) (W1)	(Ref)			
Student status (international) (W1)	−0.577	1.438	−0.401	0.689
Living situation (on-campus) (W1)	0.260	0.828	0.314	0.754
Relocated for university (yes) (W1)	0.103	0.656	0.158	0.875
Proportion of online classes (W4)	−0.211	0.206	−1.026	0.306
Engagement in university life (W4)	0.222	0.245	0.905	0.367
Belongingness (INQ) (W4)	−0.037	0.194	−0.188	0.851
Depression stigma (DSS) (W1)	−0.159	0.042	−3.753	<0.001 *

Note: * *p* > 0.05; Ref = Reference category; W1 = Wave 1; W4 = Wave 4.

**Table 4:** Logistic regression model for formal help seeking behavior at Wave 4 (*N* = 165).

	S.E.	Wald *χ*^2^	OR	*p*
Intercept	3.782	3.175	0.001	0.075
Age (in years) (W1)	0.182	0.354	1.114	0.552
Gender (female) (W1)	0.681	2.795	0.320	0.095
Gender (other/prefer not to say) (W1)	1.099	0.092	1.394	0.762
Gender (male) (W1)	(Ref)			
Student status (international) (W1)	1.510	0.013	1.185	0.910
Living situation (on-campus) (W1)	0.787	0.281	0.659	0.596
Relocated for university (W1)	0.637	4.316	3.754	0.038 *
Proportion of online classes (W4)	0.217	0.951	0.809	0.330
Engagement in university life (W4)	0.294	4.030	0.554	0.045 *
Belongingness (INQ) (W4)	0.234	0.128	0.920	0.720
Depression stigma (DSS) (W1)	0.049	0.327	1.028	0.568
Perceived knowledge of how to seek help (W4)	0.645	5.690	4.657	0.017 *
Willingness to disclose mental health problems at university (W1)	0.604	4.718	3.711	0.030 *
DQ5-General Psychological Distress (W4)	0.074	11.293	1.284	0.001 *

Note: * *p* > 0.05; Ref = Reference category; W1 = Wave 1; W4 = Wave 4.

## Data Availability

The data presented in this study are available on request from the corresponding author.

## Supplementary Materials

The following supporting information can be downloaded at: https://www.mdpi.com/article/10.3390/ijerph20010120/s1, Table S1: Measures captured at each wave.
