# Supplementary material for: Factors Associated with Engagement in University Life, and Help Seeking Attitudes and Behaviour in First Year Undergraduate Students
Source: Int J Environ Res Public Health. 2022 Dec 22;20(1):120. doi: 10.3390/ijerph20010120 (PMC9819179; doi:10.3390/ijerph20010120)
Supplement: Supplementary file 1 [file ijerph-20-00120-s001.zip › ijerph-2063610-supplementary.pdf]

Table S1. Measures captured at each wave

	Wave 1	Wave 2	Wave 3	Wave 4
Demographics (age, gender, domestic student etc).	x			
Employment	x	x	x	x
Financial stress	x	x	x	x
Responsibilities	x			
Relationship status	x			x
Degree of study	x			
Study load	x			
Study load change		x	x	x
Online classes		x	x	x
Time of semester (teaching vs not teaching period)		x	x	x
Challenges	x	x	x	x
Stigma/disclosure in a university environment	x			x
Help seeking knowledge	x			x
Mental illness diagnosis	x			
Disability (days out of role, days cut back)	x	x	x	x
Depression symptoms (PHQ-9)	x	x	x	x
GAD symptoms (GAD-7)	x	x	x	x
Social Anxiety (Social Anxiety Disorder Screener)	x	x	x	x
Distress (DQ5)	x	x	x	x
Wellbeing (WHO-5)	x	x	x	x
Alcohol consumption (AUDIT-C)	x	x	x	x
Stigma (Depression stigma scale)	x			
Loneliness (De Jong Gierveld Loneliness Scale)	x	x	x	x
Engagement with university life		x	x	x
Belonging (INQ-Belong)	x	x	x	x
Social support (Schuster Social Support Scale)	x	x	x	x
Personality (BFI-10)	x			
Academic self-efficacy (College Self-Efficacy Inventory (study and social subscales)	x	x	x	x
Functioning (Re-QoL)	x	x	x	x
Attitudes toward professional psychological treatment (ATSPPH-SF)	x			x
Help seeking (GHSQ)	x	x	x	x
COVID impacts (WSAS)	x			
Social media use (Bergen Facebook Addiction Scale)	x	x	x	x
Willingness to participate in future research				x
